# Data on the enzymatic conversion of alkaline peroxide oxidative pretreated sugarcane bagasse for the production of fermentable sugars

**DOI:** 10.1016/j.dib.2019.103867

**Published:** 2019-03-20

**Authors:** Augustine O. Ayeni, Daniel T. Oyekunle, Oluwajimi C. Shodipe, Johnson A. Folayan

**Affiliations:** a Department of Chemical Engineering, College of Engineering, Covenant University, Ota, Nigeria; b Department of Petroleum Engineering, University of Ibadan, Nigeria

**Keywords:** Fermentable sugars, Central composite design, Pretreatment, Enzymatic hydrolysis, Optimization

## Abstract

Central composite design (CCD) approach of the response surface methodology design of experiment was adopted to determine the production of fermentable sugars after enzymatic conversion of alkaline peroxide oxidative pretreated sugarcane bagasse lignocellulose. MINITAB 16 statistical software was used to design the experiments, evaluate and interpret data generated during the process. The effects of factors such as time, hydrogen peroxide concentration, and temperature on treated biomass for reducing sugars (RS) production were investigated. Operating pretreatment conditions (low–high design levels) were reaction time (6–10 h), hydrogen peroxide concentrations (1–3%v/v), and reaction temperature (60–90 °C). With the desirability of optimization of 1.000, optimal reducing sugar yield after enzymatic hydrolysis was validated to be at 100.2 °C, reaction time of 4.6 h, and hydrogen peroxide concentration of 0.3% with optimum RS yield of 153.74 mg equivalent glucose/g biomass.

**Table:** Specifications Table

Subject area	*Biotechnology*
More specific subject area	*Bioresources*
Type of data	*Table, image, figure*
How data was acquired	*MINITAB 16 statistical software was used for the experimental design, data interpretation, and optimization of the enzymatic conversion process. The optimization was evaluated on the enzyme hydrolyzed samples after the initial biomass pretreatment. Weighing scale (RADWAG XA-82/220/2X) for gravimetric analysis to determine extractive, hemicellulose, lignin, ash, and cellulose contents. Ultraviolet–Visible Spectrophotometry (JENWAY UV/VIS 6405) to quantify the fermentable sugars.*
Data format	*Raw, filtered, analyzed.*
Experimental factors	*Juice were extracted from sugar cane stalks at a local mill to obtain bagasse, the bagasse obtained was air-dried to further remove all remaining juice content. Dried sugarcane bagasse were milled for particle size reduction. Experimental factors considered are temperature, time, and hydrogen peroxide concentration, reducing sugars concentration.*
Experimental features	*Alkaline pretreatments were designed and optimized with central composite design using MINITAB 16 statistical software.*
Data source location	*Ota, Ogun state, Nigeria: 6*^*o*^*40′N 3*^*o*^*08′E*
Data accessibility	*Data is presented within this article*
Related research article	*A.O. Ayeni, M.O. Daramola, P.T. Sekoai, O. Adeeyo, M.J. Garba, A.A. Awosusi, Statistical modelling and optimization of alkaline peroxide oxidation pretreatment process on rice husk cellulosic biomass to enhance enzymatic convertibility and fermentation to ethanol. Cellulose, 25, 2018, 2487–2504*[1]*.*

**Table:** 

**Value of the data** • Data may be useful to compare similar studies using other lignocelluloses as feedstock with the prevailing experimental conditions. • Validated models generated from data can be used to predict fermentable sugar production within and outside the chosen lower and upper levels of the operating parameters. • Data can guide the usage of a small scale pilot plant for the production of reducing sugars.

## 1 Data

The compositional distribution of the raw biomass samples estimated by gravimetric method [1], [2] (Table 1) has total polysaccharide content of 62.56% (w/w) indicating sugarcane bagasse as a potential feedstock for the production of fuels and chemicals. Table 2 shows the pretreatment operating parameters for the duplicated experimental runs and the corresponding fermentable (reducing) sugars production.

**Table 1:** Dried raw sugarcane bagasse compositional analysis.

	Composition (%w/w)
Extractive	3.40
Hemicellulose	25.61
Lignin	29.84
Cellulose	36.95
Ash	4.20

**Table 2:** Design matrix and compositional estimation of biomass after pretreatment and enzymatic hydrolysis.

Run Order	Time (h)	H_2_O_2_ (%v/v)	Temperature (°C)	Observed RS yield (mg/g)	Predicted RS yield (mg/g)	Residual
1	8	0.32	75	78.23	80.64	−2.4
2	10	3	90	71.08	72.66	−1.6
3	4.64	2	75	93.07	99.04	−6.0
4	6	1	60	73.74	71.65	2.1
5	6	1	90	113.35	108.48	4.9
6	8	2	75	62.36	76.26	*−13.9*
7	8	2	49.77	68.90	69.40	−0.5
8	11.36	2	75	85.63	80.37	5.3
9	10	1	90	82.83	85.58	−2.8
10	6	3	90	93.29	93.05	0.2
11	6	3	60	87.22	83.96	3.3
12	8	2	75	78.43	76.26	2.2
13	8	2	75	81.27	76.26	5.0
14	8	3.68	75	81.83	80.13	1.7
15	10	1	60	70.11	69.84	0.3
16	8	2	100.23	90.07	90.28	−0.2
17	8	2	75	82.61	76.26	6.3
18	10	3	60	80.31	84.67	−4.4
19	8	2	75	78.00	76.26	1.7
20	8	2	75	75.03	76.26	−1.2

**Fig. 1:**
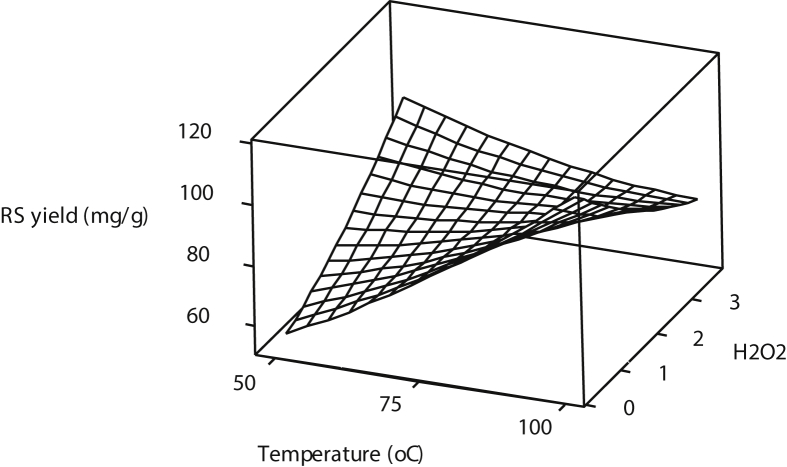
Surface plot for reducing sugar yield against %H_2_O_2_ and Temperature.

**Fig. 2:**
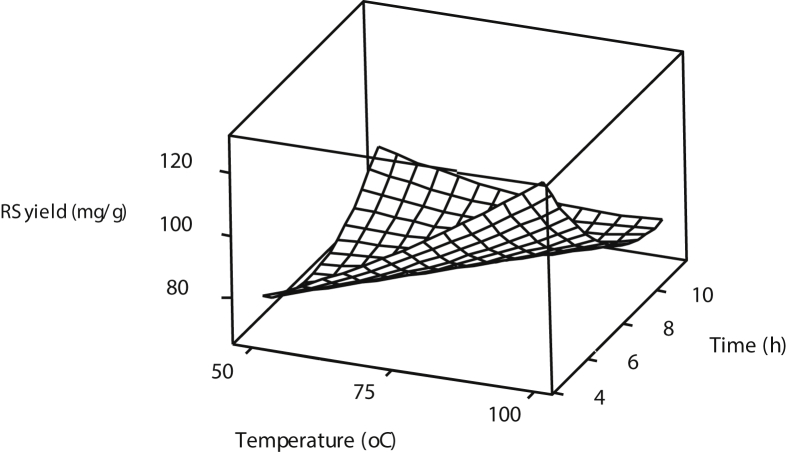
Surface plot for reducing sugar yield against Time and Temperature.

**Fig. 3:**
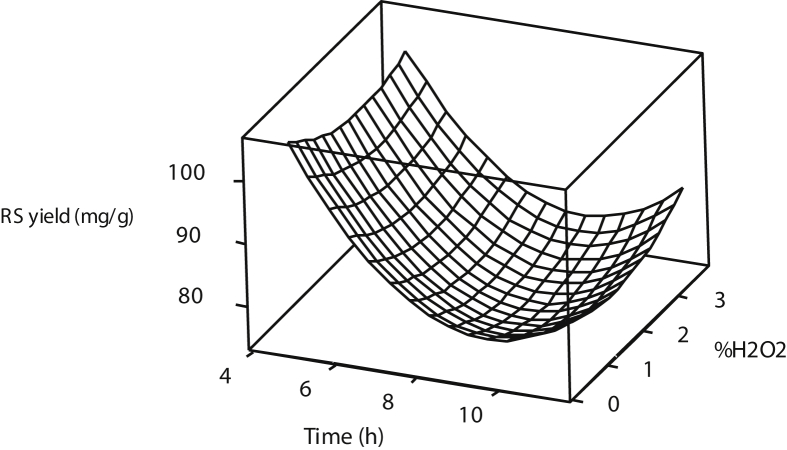
Surface plot for reducing sugar yield against %H_2_O_2_ and Time.

**Fig. 4:**
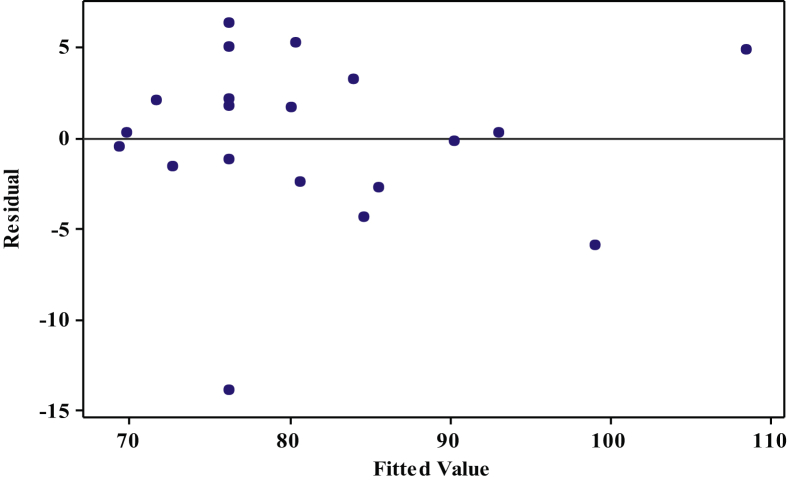
Diagnosis for the regression model fit for the experimental and predicted values.

Fig. 1, Fig. 2, Fig. 3 show the interactions of pretreatment operating parameters on reducing sugars (RS) yields. The surface plots showed effective range prediction for the optimum production of fermentable sugars. Fig. 4 shows the experimental data and the predicted values having minimal deviations.

Table 3 shows the analysis of variance (ANOVA) description of generated regression model (Eqs. (1), (2)) used for the adequate prediction of reducing sugars with the operating pretreatment parameters values.

**Table 3:** Analysis of Variance (ANOVA) for the regression model obtained from the central composite design.

Source	DF	Seq SS	Adj SS	Adj MS	*F*	*P*
Regression	9	3808.65	3808.65	423.183	15.56	0.000
Linear	3	1894.18	582.85	194.283	7.14	0.001
X_1_	1	841.18	83.73	83.729	3.08	0.090
X_2_	1	0.63	217.13	217.130	7.98	0.008
X_3_	1	1052.36	170.93	170.930	6.28	0.018
Square	3	693.74	693.74	231.247	8.50	0.000
X_1_^2^	1	595.85	650.82	650.817	23.92	0.000
X_2_^2^	1	51.73	61.33	61.327	2.25	0.144
X_3_^2^	1	46.17	46.17	46.169	1.70	0.203
Interaction	3	1220.73	1220.73	406.910	14.96	0.000
X_1_X_2_	1	6.34	6.34	6.343	0.23	0.633
X_1_X_3_	1	444.85	444.85	444.851	16.35	0.000
X_2_X_3_	1	769.54	769.54	769.535	28.29	0.000
Residual error	30	816.10	816.10	27.203		
Lack-of-fit	5	280.16	280.16	56.032	2.61	0.049
Pure error	25	535.94	535.94	21.438		
Total	39	4624.75				

**Fig. 5:**
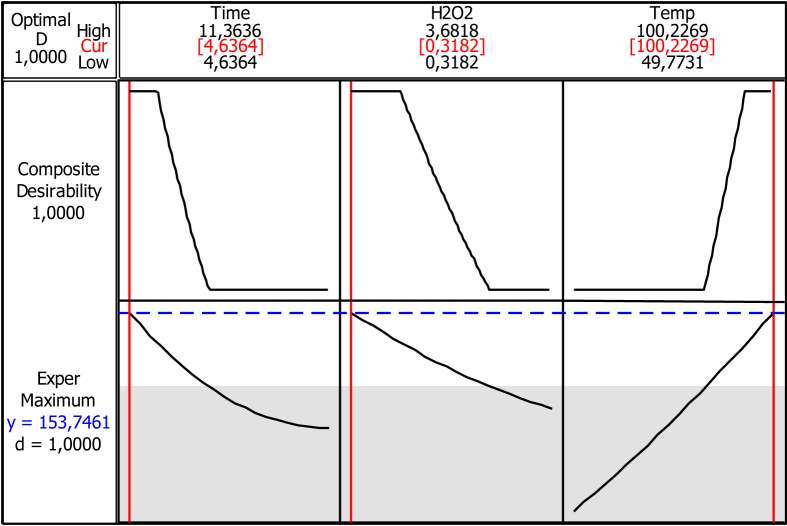
RS yield at optimized operating pretreatment conditions.

Fig. 5 shows the validated optimum pretreatment conditions (Time: 4.63 h, H_2_O_2_: 0.32%v/v, and Temperature: 100.23 °C) and the optimum value of RS (153.75 mg/g).

## 2 Experimental design, materials, and methods

### 2.1 Enzymatic conversion of treated biomass to fermentable sugars

Experimental data generated after enzymatic hydrolysis of treated biomass were used to develop a regression model for predicting the reducing sugar yields for different operating parameters. After each pretreatment, mass loss was estimated for required mass balances for enzymatic hydrolysis step. The cellulase enzyme complex (*Trichoderma reesei*) having an activity of 57.8 FPU/ml was used on 2% biomass loading and 25 FPU/g biomass enzyme dose. A cost effective method for hydrolyzing cellulose and hemicellulose to valuable products such as fermentable sugars is through the enzymatic hydrolysis process [3].

### 2.2 Model development, optimization, and validation of the optimum conditions

A model equation was generated from the experimental data by considering reducing sugar (Y) yield as the predicted response associated with factor combinations of time (X_1_), %H_2_O_2_ (X_2_), and temperature (X_3_).

$\alpha_{1}$ to $\alpha_{2,3}$ (Eq. 1) are the coefficients to be estimated from regression representing linear, quadratic, and the interactive effects. The regression analysis, plotting of response surfaces, generating predicted responses of RS yields, and the optimization step were executed using MINITAB 16.

(1) $$Y=\alpha_{1}+\alpha_{1}X_{1}+\alpha_{2}X_{2}+\alpha_{3}X_{3}+\alpha_{1,1}X_{1}^{2}+\alpha_{2,2}X_{2}^{2}+\alpha_{3,3}X_{3}^{2}+\alpha_{1,2}X_{1}X_{2}+\alpha_{1,3}X_{1}X_{3}+\alpha_{2,3}X_{2}X_{3}$$

The regression model relating the RS yields (Y) to the operating variables of time, H_2_O_2_, and temperature (X_3_) can be written as follows:

(2) $$Y=11.4620-9.2296X_{1}+26.1702X_{2}+1.9008X_{3}+1.1880X_{1}^{2}+1.4587X_{2}^{2}+0.0056X_{3}^{2}+0.3148X_{1}X_{2}-0.1758X_{1}X_{3}-0.4623X_{2}X_{3}$$

*R*^*2*^ = 82.35%; *R*^*2*^ *(pred)* = 73.14%; *R*^*2*^ *(adj)* = 77.06%. *PRESS* = 1242.24. *S* = 5.22

Substituting the values of the operating parameters into Eq. 2 gives the predicted values for the RS yields (Table 2). Fig. 4 shows the accuracy of symmetry of the residuals (absolute difference between experimental and predicted values) versus the fitted values indicating the reliability of Eq. 2 for adequate prediction.

Data obtained using the analysis of variance (Table 3) validate the reliability of regression model by interpreting with the *F-*statistics and the probability values (*P*-values) of the linear, quadratic, and interactive effects on RS yields. The desirability of optimization was 1.000, optimal reducing sugar yield of 153.74 mg/g after pretreatment was validated to be at 100.2 °C, reaction time of 4.6 h, and hydrogen peroxide concentration of 0.3% (Fig. 5). Factors that affect the yield of reducing sugar from lignocelluloses include accessibility and adsorption characteristics of the cellulose, reactivity of the cellulose and adsorption characteristics of the lignin present [4], [5].
