# Supplementary material for: Data on the enzymatic conversion of alkaline peroxide oxidative pretreated sugarcane bagasse for the production of fermentable sugars
Source: Data Brief. 2019 Mar 20;24:103867. doi: 10.1016/j.dib.2019.103867 (PMC6441737; doi:10.1016/j.dib.2019.103867)
Supplement: Multimedia component 2 [file mmc2.docx]

**Response Surface Regression: Exper versus Time; H2O2; Temp**

The analysis was done using uncoded units.

Estimated Regression Coefficients for Exper

Term Coef SE Coef T P

Constant 11,4620 43,4603 0,264 0,794

Time -9,2296 5,2609 -1,754 0,090

H2O2 26,1702 9,2631 2,825 0,008

Temp 1,9008 0,7583 2,507 0,018

Time*Time 1,1880 0,2429 4,891 0,000

H2O2*H2O2 1,4587 0,9715 1,501 0,144

Temp*Temp 0,0056 0,0043 1,303 0,203

Time*H2O2 0,3148 0,6520 0,483 0,633

Time*Temp -0,1758 0,0435 -4,044 0,000

H2O2*Temp -0,4623 0,0869 -5,319 0,000

S = 5,21569 PRESS = 1242,24

R-Sq = 82,35% R-Sq(pred) = 73,14% R-Sq(adj) = 77,06%

Analysis of Variance for Exper

Source DF Seq SS Adj SS Adj MS F P

Regression 9 3808,65 3808,65 423,183 15,56 0,000

Linear 3 1894,18 582,85 194,283 7,14 0,001

Time 1 841,18 83,73 83,729 3,08 0,090

H2O2 1 0,63 217,13 217,130 7,98 0,008

Temp 1 1052,36 170,93 170,930 6,28 0,018

Square 3 693,74 693,74 231,247 8,50 0,000

Time*Time 1 595,85 650,82 650,817 23,92 0,000

H2O2*H2O2 1 51,73 61,33 61,327 2,25 0,144

Temp*Temp 1 46,17 46,17 46,169 1,70 0,203

Interaction 3 1220,73 1220,73 406,910 14,96 0,000

Time*H2O2 1 6,34 6,34 6,343 0,23 0,633

Time*Temp 1 444,85 444,85 444,851 16,35 0,000

H2O2*Temp 1 769,54 769,54 769,535 28,29 0,000

Residual Error 30 816,10 816,10 27,203

Lack-of-Fit 5 280,16 280,16 56,032 2,61 0,049

Pure Error 25 535,94 535,94 21,438

Total 39 4624,75

Obs StdOrder Exper Fit SE Fit Residual St Resid

1 11 78,232 80,643 2,874 -2,411 -0,55

2 8 71,078 72,665 3,018 -1,587 -0,37

3 9 93,068 99,035 2,874 -5,967 -1,37

4 1 73,743 71,654 3,018 2,089 0,49

5 5 113,346 108,484 3,018 4,862 1,14

6 18 62,355 76,262 1,504 -13,907 -2,78 R

7 13 68,901 69,402 2,874 -0,501 -0,12

8 10 85,626 80,369 2,874 5,257 1,21

9 6 82,827 85,580 3,018 -2,753 -0,65

10 7 93,287 93,050 3,018 0,237 0,06

11 3 87,216 83,961 3,018 3,255 0,77

12 20 78,429 76,262 1,504 2,167 0,43

13 17 81,265 76,262 1,504 5,003 1,00

14 12 81,833 80,131 2,874 1,702 0,39

15 2 70,107 69,842 3,018 0,265 0,06

16 14 90,072 90,281 2,874 -0,209 -0,05

17 19 82,611 76,262 1,504 6,349 1,27

18 4 80,307 84,667 3,018 -4,360 -1,02

19 15 78,000 76,262 1,504 1,738 0,35

20 16 75,032 76,262 1,504 -1,230 -0,25

21 11 78,232 80,643 2,874 -2,411 -0,55

22 8 71,078 72,665 3,018 -1,587 -0,37

23 9 93,068 99,035 2,874 -5,967 -1,37

24 1 73,743 71,654 3,018 2,089 0,49

25 5 113,346 108,484 3,018 4,862 1,14

26 18 62,355 76,262 1,504 -13,907 -2,78 R

27 13 68,901 69,402 2,874 -0,501 -0,12

28 10 85,626 80,369 2,874 5,257 1,21

29 6 82,827 85,580 3,018 -2,753 -0,65

30 7 93,287 93,050 3,018 0,237 0,06

31 3 87,216 83,961 3,018 3,255 0,77

32 20 78,429 76,262 1,504 2,167 0,43

33 17 81,265 76,262 1,504 5,003 1,00

34 12 81,833 80,131 2,874 1,702 0,39

35 2 70,107 69,842 3,018 0,265 0,06

36 14 90,072 90,281 2,874 -0,209 -0,05

37 19 82,611 76,262 1,504 6,349 1,27

38 4 80,307 84,667 3,018 -4,360 -1,02

39 15 78,000 76,262 1,504 1,738 0,35

40 16 75,032 76,262 1,504 -1,230 -0,25

R denotes an observation with a large standardized residual.

Predicted Response for New Design Points Using Model for Exper

Point Fit SE Fit 95% CI 95% PI

1 80,643 2,87408 ( 74,774; 86,513) (68,4814; 92,806)

2 72,665 3,01828 ( 66,501; 78,829) (60,3581; 84,972)

3 99,035 2,87408 ( 93,165; 104,905) (86,8731; 111,197)

4 71,654 3,01828 ( 65,490; 77,818) (59,3472; 83,961)

5 108,484 3,01828 (102,320; 114,649) (96,1775; 120,791)

6 76,262 1,50417 ( 73,190; 79,334) (65,1757; 87,348)

7 69,402 2,87408 ( 63,533; 75,272) (57,2403; 81,564)

8 80,369 2,87408 ( 74,499; 86,238) (68,2068; 92,531)

9 85,580 3,01828 ( 79,416; 91,745) (73,2735; 97,887)

10 93,050 3,01828 ( 86,886; 99,215) (80,7436; 105,357)

11 83,961 3,01828 ( 77,796; 90,125) (71,6538; 96,267)

12 76,262 1,50417 ( 73,190; 79,334) (65,1757; 87,348)

13 76,262 1,50417 ( 73,190; 79,334) (65,1757; 87,348)

14 80,131 2,87408 ( 74,262; 86,001) (67,9694; 92,294)

15 69,842 3,01828 ( 63,677; 76,006) (57,5347; 82,148)

16 90,281 2,87408 ( 84,411; 96,150) (78,1186; 102,443)

17 76,262 1,50417 ( 73,190; 79,334) (65,1757; 87,348)

18 84,667 3,01828 ( 78,502; 90,831) (72,3597; 96,973)

19 76,262 1,50417 ( 73,190; 79,334) (65,1757; 87,348)

20 76,262 1,50417 ( 73,190; 79,334) (65,1757; 87,348)

21 80,643 2,87408 ( 74,774; 86,513) (68,4814; 92,806)

22 72,665 3,01828 ( 66,501; 78,829) (60,3581; 84,972)

23 99,035 2,87408 ( 93,165; 104,905) (86,8731; 111,197)

24 71,654 3,01828 ( 65,490; 77,818) (59,3472; 83,961)

25 108,484 3,01828 (102,320; 114,649) (96,1775; 120,791)

26 76,262 1,50417 ( 73,190; 79,334) (65,1757; 87,348)

27 69,402 2,87408 ( 63,533; 75,272) (57,2403; 81,564)

28 80,369 2,87408 ( 74,499; 86,238) (68,2068; 92,531)

29 85,580 3,01828 ( 79,416; 91,745) (73,2735; 97,887)

30 93,050 3,01828 ( 86,886; 99,215) (80,7436; 105,357)

31 83,961 3,01828 ( 77,796; 90,125) (71,6538; 96,267)

32 76,262 1,50417 ( 73,190; 79,334) (65,1757; 87,348)

33 76,262 1,50417 ( 73,190; 79,334) (65,1757; 87,348)

34 80,131 2,87408 ( 74,262; 86,001) (67,9694; 92,294)

35 69,842 3,01828 ( 63,677; 76,006) (57,5347; 82,148)

36 90,281 2,87408 ( 84,411; 96,150) (78,1186; 102,443)H_2_O_2_

37 76,262 1,50417 ( 73,190; 79,334) (65,1757; 87,348)

38 84,667 3,01828 ( 78,502; 90,831) (72,3597; 96,973)

39 76,262 1,50417 ( 73,190; 79,334) (65,1757; 87,348)

40 76,262 1,50417 ( 73,190; 79,334) (65,1757; 87,348)

Normal probability plot

|  |  |  |  |
| --- | --- | --- | --- |
|  |  |  |  |
